# Supplementary material for: Hippocampal Commissural Circuitry Shows Asymmetric cAMP-Dependent Synaptic Plasticity
Source: ACS Chem Neurosci. 2025 Oct 13;16(21):4236–45. doi: 10.1021/acschemneuro.5c00454 (PMC12593395; doi:10.1021/acschemneuro.5c00454)
Supplement: Supplementary file 1 [file cn5c00454_si_001.pdf]

Hippocampal Commissural Circuitry Shows Asymmetric cAMP-Dependent Synaptic Plasticity

Lukas Faiss [1,2] (https://orcid.org/0000-0001-5150-5541), lukas.faiss@dzne.de
Aikaterini Salivara [1,2], aikaterini.salivara@dzne.de
Silvia Oldani [4] (https://orcid.org/0000-0002-9034-6237), silvia.oldani@ymail.com
Jörg Breustedt [2] (https://orcid.org/0000-0003-4248-9919), joerg.breustedt@charite.de
Dietmar Schmitz [1,2,3,†] (https://orcid.org/0000-0003-2741-5241), dietmar.schmitz@charite.de
Benjamin R. Rost [1,2,†] (https://orcid.org/0000-0003-1906-0081), benjamin.rost@dzne.de

[1] German Center for Neurodegenerative Diseases (DZNE), 10117 Berlin, Germany.
[2] Institute of Cell Biology and Neurobiology, and Neuroscience Research Center, Charité-Universitätsmedizin Berlin, corporate member of Freie Universität Berlin and Humboldt-Universität zu Berlin, 10117 Berlin, Germany.
[3] Bernstein Center for Computational Neuroscience, Humboldt Universität zu Berlin, 10115 Berlin, Germany.
[4] Maxwell Biosystems, Albisriederstrasse 253, 8047 Zürich, Switzerland
[†]Correspondence

This file contains three supplemental figures providing supporting information for the manuscript. The figures illustrate correlations of age, sex, and release probability with forskolin (FSK) responsiveness, schemata of expression patterns in slices exhibiting FSK effects and no effects, and correlations between contralateral and ipsilateral recordings.

Supplemental Figures

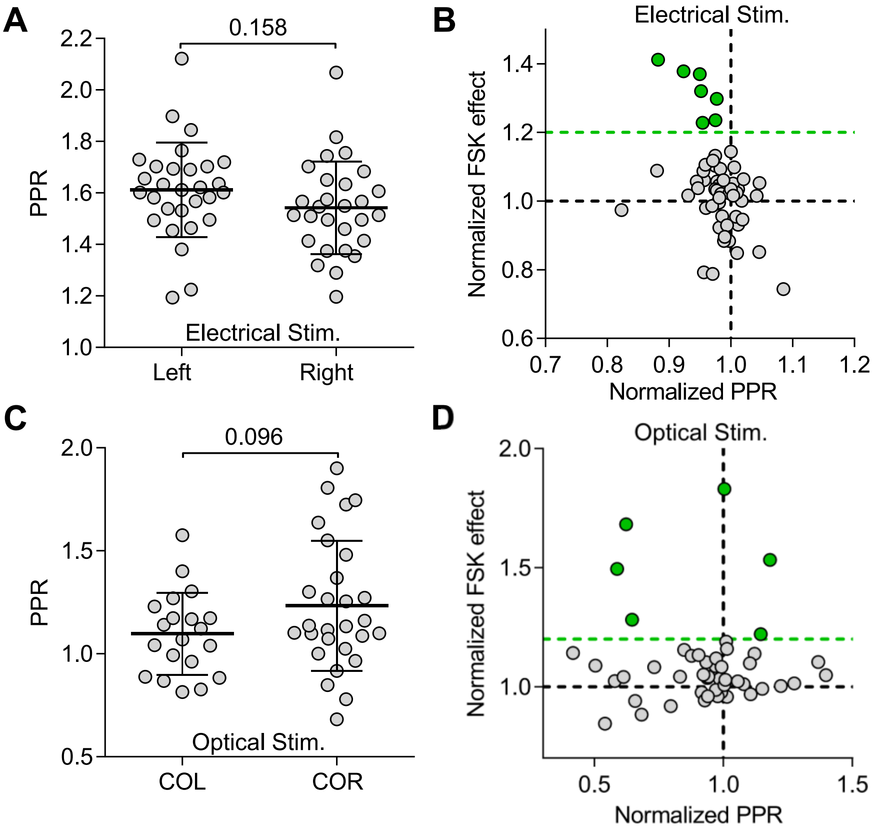

Supplemental Figure 1. Release probability across hemispheres and correlation with FSK responsiveness in WT animals. A Release probability of electrically-evoked baseline recordings from the left and right hemispheres. Bars represent PPR during the 10-minute baseline period (Left: 1.61 ± 0.18, 95% CI [1.54, 1.68], n = 29, N = 15; Right: 1.54 ± 0.18, 95% CI [1.47, 1.61], n = 28, N = 15). No significant difference was detected between hemispheres (unpaired t-test, p = 0.158). **B** Correlation plot of normalized PPR (electrical recordings from both hemispheres) versus normalized FSK effect. Green data points (n = 7) indicate FSK effect sizes above the 20% threshold (green dashed line). The black dashed line denotes the division between paired-pulse depression (< 1) and facilitation (> 1). All recordings with detectable FSK effects exhibited paired-pulse depression, suggesting a link between increased fEPSP amplitude and increased release probability. **C** Release probability of optically stimulated recordings from WT COL and COR inputs. Bars represent PPR during the 10-minute baseline period (COL: 1.09 ± 0.20, 95% CI [1.00, 1.19], n = 20, N = 5; COR: 1.23 ± 0.32, 95% CI [1.11, 1.36], n = 28, N = 11). No significant difference was detected (unpaired t-test, p = 0.096). **D** Correlation plot as in **B**, but for optically stimulated COL and COR recordings. No correlation was observed between normalized PPR and FSK induced increases in fEPSP amplitude.

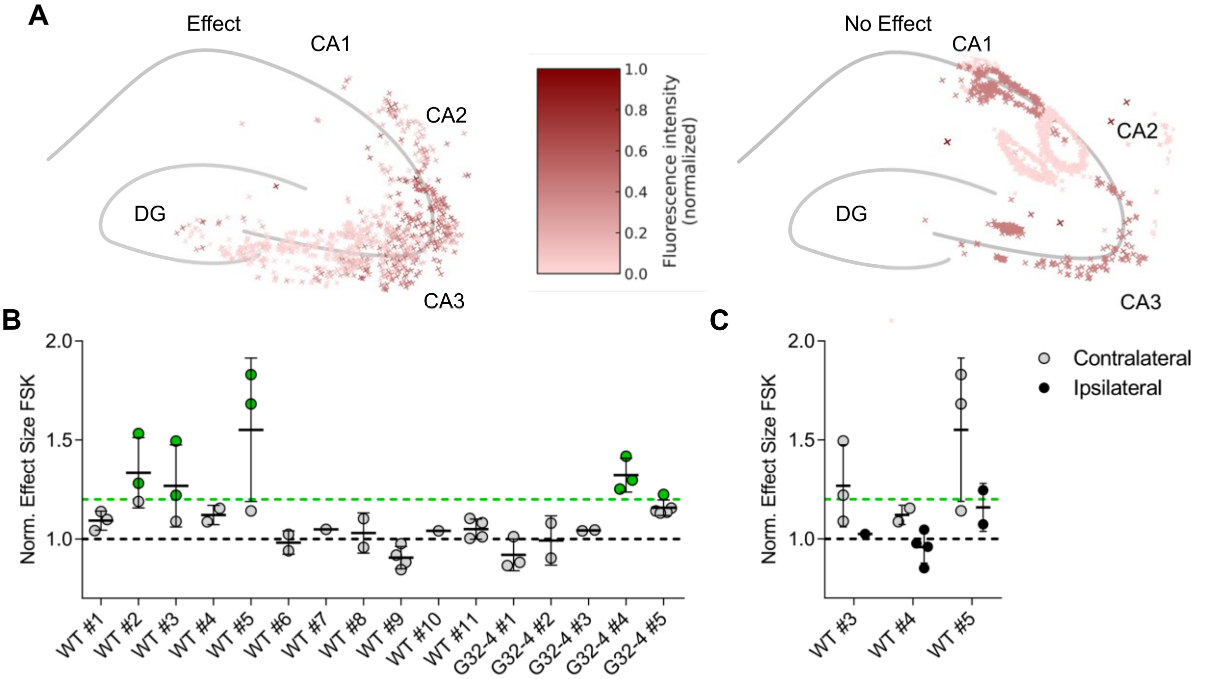

Supplemental Figure 2. Variable viral expression in CA3 and individual display of forskolin effects detailed break-down. A Expression pattern of ChrimsonR-positive cells in the dorsal hippocampus from animals that exhibited strong FSK effects (left, N = 3) and no FSK effects (right, N = 8). Fluorescent somata were detected from red-channel images, normalized, thresholded, and plotted as points scaled by intensity. **B** Summary of COR effects in WT and G32-4 Cre recordings, grouped by animal. **C** Comparison of contralateral and ipsilateral recordings from the same WT animal.

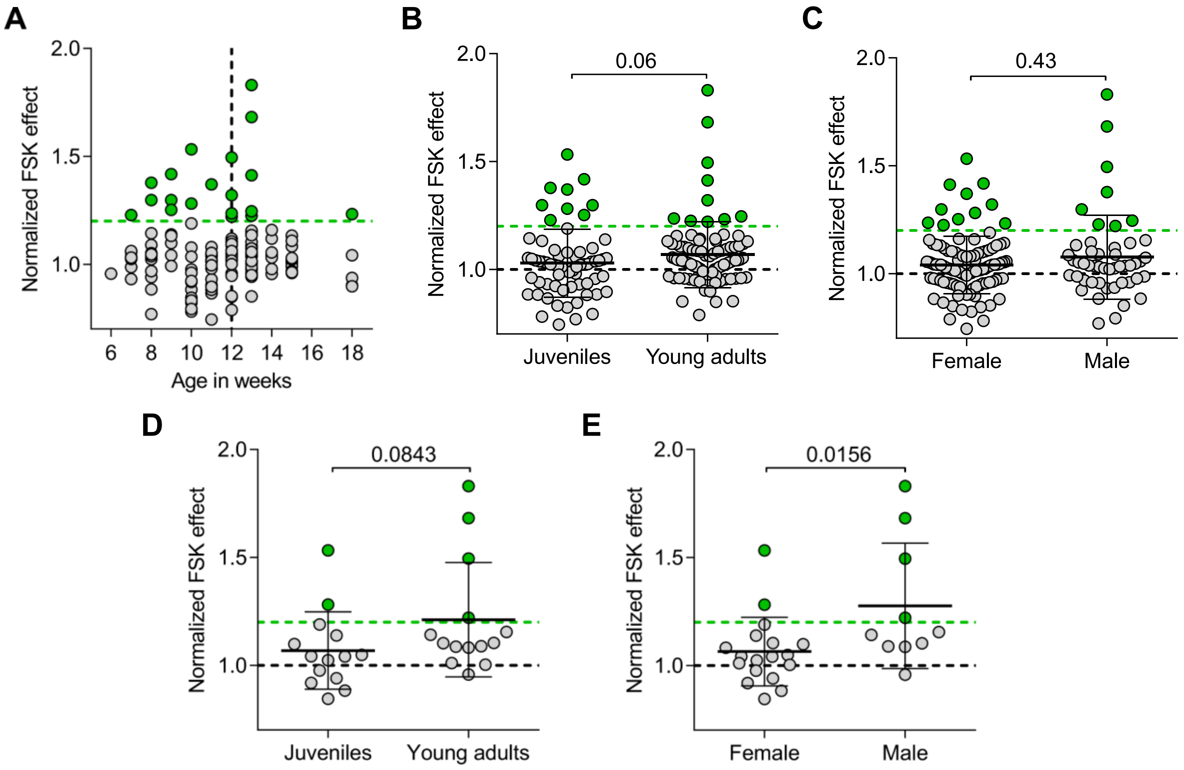

Supplemental Figure 3. No correlation of age or sex with FSK responsiveness. **A** Correlation plot of age versus normalized FSK effect, pooling all recordings in this study (n = 157). Green data points (n = 19) indicate effect sizes exceeding the 20% threshold (green dashed line). The black dashed line marks the boundary between juvenile and young adult animals (12 weeks). FSK responsiveness did not correlate with animal age. **B** Comparison of FSK effect size between juvenile and young adult animals, across all recordings. Bars represent mean fEPSP amplitude during the 10-minute post-FSK period, normalized to baseline (Juveniles: 102.9 ± 15.8%, 95% CI [98.9%, 106.8%], n = 66, N = 19; Young adults: 106.7 ± 15.3%, 95% CI [103.5%, 109.8%], n = 91, N = 26). No significant difference was observed (Mann–Whitney test, p = 0.06). **C** Comparison of FSK effect size between female and male animals, across all recordings. Bars represent mean normalized fEPSP amplitude during the 10-minute post-FSK period (Female: 104.0 ± 13.3%, 95% CI [101.5%, 106.6%], n = 108, N = 31; Male: 107.7 ± 19.5%, 95% CI [102.1%, 113.3%], n = 49, N = 14). No significant difference was observed (Mann–Whitney test, p = 0.43). **D** Comparison of FSK effect size between juvenile and young adult animals of WT COR recordings (Juveniles: 106.8 ± 17.9%, 95% CI [96.5%, 117.2%], n = 14, N = 5; Young adults: 121.1 ± 26.5%, 95% CI [105.8, 109.8], n = 14, N = 6). No significant difference was observed (Mann–Whitney test, p = 0.08). **E** Comparison of FSK effect size between female and male animals of WT COR recordings (Female: 106.4 ± 15.8%, 95% CI [98.5%, 114.3%], n = 18, N = 7; Male: 127.6 ± 29.0%, 95% CI [106.8%, 148.3%], n = 10, N = 4). A significant difference was observed (Mann–Whitney test, p = 0.015).
